# Characteristics of men who have sex with men (MSM) who tested for HIV while donating blood in Latvia

**DOI:** 10.1186/1471-2334-14-S2-P10

**Published:** 2014-05-23

**Authors:** A Mozalevskis, A Karnite

**Affiliations:** 1 National Center of Epidemiology (CNE), Salud Carlos III Institute, Madrid, Spain; 2 Riga Stradins University, Department of Public Health and Epidemiology, Riga, Latvia

## Background

In 2005, Latvia lifted the ban on blood donation for men who have sex with men (MSM). The prevalence of HIV infection in blood donations since then has been stable (around 0.02%), though it is higher than the European average (0.009% in 2006). We aim at describing the MSM who report that their last HIV test was done while donating blood (WDB) to approach the role of MSM in the donor blood safety.

## Methods

We used the Latvian dataset from the European MSM Internet Survey (EMIS, 2010). We compared the MSM who had their last HIV test WDB with those who had their last HIV test elsewhere in terms of socio-demographic and behavioral factors using Chi-square and Fisher's exact tests.

## Results

Of 708 EMIS respondents in Latvia, 346 (49.8%) ever tested for HIV and of these, 46 (13.3%) received their last HIV test WDB. Reactive HIV test were lower among MSM who reported having last test WDB than elsewhere (4% vs. 8% p=0.292). Respondents below 25 years, MSM self-identified as bisexual rather than gay and MSM who were less open about their sexual orientation were more likely to have their last HIV test WDB (25% vs. 11%, p=0.003; 22% vs. 11%, p=0.066; 15% vs. 5.5% p=0.061, respectively). There was no difference in terms of reported risk behavior. Only one respondent who received last HIV test WDB (2%) reported having the opportunity to discuss sexual practices before the test while they were 48 (16%) among those tested elsewhere (p=0.047).

## Conclusion

MSM donate blood and the prevalence of HIV infection in blood donation from MSM might be higher than from general population. MSM who donate blood are younger, self-identified as bisexual and not open about their sexual orientation. More attention is needed to evaluate their sexual risk behaviors in a sensitive and acceptable way during the pre-donation interview.

